# Editorial: Insights in epigenomics and epigenetics: 2022

**DOI:** 10.3389/fgene.2023.1205975

**Published:** 2023-06-13

**Authors:** Douglas M. Ruden, Mojgan Rastegar

**Affiliations:** ^1^ C. S. Mott Center for Human Health and Development, Department of Obstetrics and Gynecology, Institute of Environmental Health Sciences, Wayne State University, Detroit, MI, United States; ^2^Department of Biochemistry and Medical Genetics, Max Rady College of Medicine, Rady Faculty of Health Sciences, University of Manitoba, Winnipeg, MB, Canada

**Keywords:** telomeres, 5mC, M6A, histone chaperones, epigenetics, epigenomics

## 1 Overview: The 4 original research papers in this research topic

The top-most viewed original research paper in this “Insights” Research Topic was by Wang and colleagues and was titled “Genetic regulation of newborn telomere length is mediated and modified by DNA methylation” (Wang et al.). This paper came out in October, 2022, and already has over 1,167 views at the time this editorial was written, according to the Frontiers Media site “Loop Research Network” which maps “views,” “downloads,” “citations,” “social buzz,” and “demographics” (https://loop.frontiersin.org/). This research paper studied 281 mom-newborn pairs from the ENVIRonmental influence ON early AGEing (ENVIRONAGE) pregnancy cohort comparing DNA 5 mC methylation and telomere (TEL) lengths on genomic DNA isolated from the blood of the mother with that from the babies’ umbilical cord. This paper was a tour-de-force of high-dimensional omics analyses.

The second-most viewed original research paper was by Lothion-Roy and colleagues and is titled, “Clinical and molecular significance of the RNA m6A methyltransferase complex in prostate cancer” (Lothion-Roy et al.). This paper came out in January, 2023, and already has 1,096 views. N6-methyl adenine (m6A) is the most abundant internal mRNA modification in eukaryotes and regulates processes such as mRNA translation into protein, mRNA alternative splicing, and mRNA stability. The authors studied the levels of key proteins in the m6A pathway in prostate cancer cells and cell lines. Prostate cancer is the most abundant non-cutaneous cancer in men and the authors wanted to determine whether any of the m6A proteins correlate with the type of prostate cancer or the severity and prognosis of the disease. Overall, this is an exciting paper which points to m6A biology as a new avenue for prostate cancer research.

The third-most viewed original research paper was by Thumfart and colleagues and was titled “Long-term effects of early postnatal stress on Sertoli cells” (Thumfart et al.). This paper also came out in October, 2022, and has 877 views in this short period of time. This paper studied Sertoli cells from adult male mice, which are tightly associated with germ cells, provide structural support for differentiating sperm, and help form a tight blood-testis barrier. The Sertoli cells are highly glycolytic because they need to provide high levels of lactate to the differentiating sperm as an energy source. The stress applied to the moms and pups was during postnatal days 1–14 following the MSUS (maternal separation combined with unpredictable maternal stress) protocol in which the moms were forced to swim in cold water for 3 h per day at random times. The most interesting finding was that multiple components of the, ETC (electron transport chain) in the mitochondria were down regulated at the protein level, causing dysregulation of the mitochondria.

The fourth-most viewed original research paper was by Li and colleagues and is titled “Whole-genome DNA methylome analysis of different developmental stages of the entomopathogenic fungus *Beauveria bassiana* NCHU-157 by nanopore sequencing” (Li et al.). This paper came out in January, 2023, and has 855 views in less than 3 months. The fungus *B. bassiana* infects the caterpillars of the moth *Spodoptera litera*, commonly known as the tobacco cutworm or the cotton leafworm, which is a major pest in several Asian countries. Therefore, understanding the basic biology and developmental epigenomics of this fungus could help in controlling the pest *S. litera*. Overall, this is an impressive demonstration of understanding the 5 mC-DNA methylome of an organism.

## 2 Future perspectives

**FIGURE 1:**
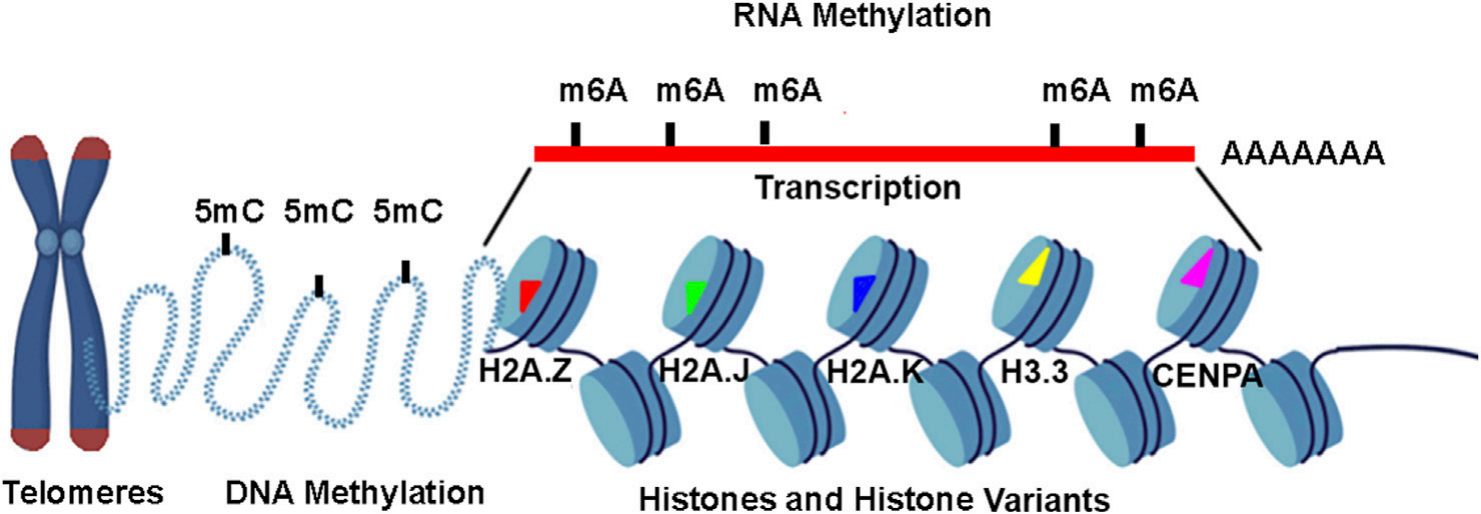
Graphical Overview of Epigenomics Themes in this Research Topic. One paper investigates how 5 mC-DNA methylation affects telomere (TEL) length in newborns. Another study measures 5 mC levels in a fungus by nanpopore sequencing. A third study investigates m6A RNA modification machinery in prostate cancer. One review discusses histone variants, such as H2A.Z, H2A.J, H2A.K, H3.3, and CENPE and their chaperones in chromatin regulation, cellular senescence and aging (Torres-Arciga et al.). Finally, a review discusses how 5 mC levels and other epigenetic marks can be used to stratify 12 or more types of medulloblastoma (Gorini et al.).

The 6 papers – 4 original research and 2 reviews – of this “Insights” Research Topic are very diverse, from histone variants in aging cells to 5 mC-levels in the various stages of fungal development, yet the common theme is that global epigenomics techniques will provide new insights into most biological phenomena (Figure 1). The field of epigenomics is cross-fertilizing and this synopsis will hopefully spur new directions in epigenomics research. For example, the histone variant review (Torres-Arciga et al.) and the studies on aging and senescence might spur new research on the role of histone variants in fungal development (Li et al.). Similarly, the ambitious use of nanopore DNA sequencing to identify global 5 mC levels during fungal development (Li et al.) might spur researchers to use nanopore DNA sequencing to characterize the epigenetic states of medulloblastoma cells (Gorini et al.). There is an unlimited number of permutations such as these that can be done, all of which could potentially open new areas of investigation and clinical management of diseases in humans, animals, plants, and even fungi.

